# Limited Attention to Climate Change in U.S. Sociology

**DOI:** 10.1007/s12108-024-09624-4

**Published:** 2024-06-07

**Authors:** Sofia Hiltner

**Affiliations:** 1 Department of Sociology, University of Michigan, Ann Arbor MI, USA

**Keywords:** Climate Change, Environment, Sociology Journals, Conferences, Teaching

## Abstract

Climate change is increasingly recognized as not only a biophysical and technological problem but also a social one. Nonetheless, sociologists have expressed concern that sociology has paid relatively little attention to climate change. This deficit threatens to limit the frames available to understand and imagine solutions to the climate crisis. In this paper I report the most up-to-date and expansive empirical assessment of attention to climate change in sociology in the United States (U.S.). I find little to no mention of climate change across leading sociology journal articles (0.89%), conference sessions (1.5%), and faculty biographies (2.8%) and course listings (0.2%) in the 20 top-ranked departments in the U.S. Two leading journals, the *American Sociological Review* and *American Journal of Sociology*, have cumulatively published just three articles focused on climate change to date. This level of disciplinary attention appears low compared to the field’s engagement with other important social problems. My findings thus suggest that climate silence is persistent and pervasive in U.S. sociology. I discuss the implications of this silence and outline opportunities for sociologists, funders, journalists, and policymakers to embrace social science perspectives in climate change teaching, research, and policymaking.

## Introduction

Climate change is increasingly recognized as not only a biophysical and technological problem but also a social one (Stoddard et al., 2021). Yet the social sciences are dramatically underrepresented compared to the natural sciences in climate change research and global climate assessments such as those by the Intergovernmental Panel on Climate Change (IPCC) (Corbera et al., 2016; Overland & Sovacool, 2020; Victor, 2015). This absence threatens to limit the frames available to understand and imagine solutions to the climate crisis. One possible contributor to this phenomenon is a lack of engagement with climate change in the social sciences themselves. In this paper, I report the most up-to-date and expansive empirical overview of attention to climate change in one especially relevant social science discipline in the U.S.: sociology. Of the social sciences, sociology can uniquely attend to questions about how social structures contribute to climate change, its effects on society, and societal responses (or lack thereof) (Norgaard, 2018).

As early as 2008, with the publication of Constance Lever-Tracy’s “Global Warming and Sociology,” sociologists around the world have called for more climate change research and teaching in sociology (Liu & Szasz, 2019; Lockie, 2022; Norgaard, 2018; Pelton, 2022; Urry, 2009; Yearley, 2009; cf. Grundmann & Stehr, 2010).^1^ Though climate change has appeared to be peripheral to the discipline at large, this topic spans multiple sociological subfields and is a critical—even existential—topic for sociologists (Dietz et al., 2020; Klinenberg et al., 2020).^2^

The apparent lack of attention to climate change in sociology is particularly peculiar and problematic for several reasons.^3^ First, climate change is not new. Climate scientists, lawmakers, journalists, and activists have been aware of it for over 50 years (Bell, 2021). For example, climate scientist James Hansen testified about global warming before the U.S. Congress in 1988; activist Bill McKibben published the first popular treatise on the topic, *The End of Nature*, in 1989; and the IPCC published its first assessment in 1990. Typical lags in academic research and publication cannot fully explain the lack of engagement. Second, among social problems, climate change poses a uniquely existential threat to society that will dramatically change how humans live. Dunlap and Brulle (2015) describe how climate change is “associated with increases in ‘natural’ disasters; precipitously shifting weather patterns; threats to availability of potable water, food, and shelter; shifts in the range and prevalence of disease; species extinction; and the destabilization of ecosystems on which we depend,” with impacts including “conflicts over natural resources, social destabilization, population migration, and extensive adverse health consequences” (p. 1) (also see Kolbert, 2014; McKibben, 2012; Wallace-Wells, 2017). Third, a lack of sociological input threatens to limit understanding of and responses to climate change as a social problem, as well as threatens sociology’s relevance to human welfare (Otto et al., 2020; Norgaard, 2018; Stoddard et al., 2021). Finally, as Thierry et al. (2023) point out, climate change will destabilize society and thus higher education itself, as “there is no research on a dead planet.”

Scholars have previously examined the extent to which sociology has investigated climate change in the U.S. and elsewhere by tracking publication rates and coverage in introductory sociology courses. To assess the current status of engagement in the U.S., I extend this work by bringing it up to date and examining a wider range of sociology forums. I examine rates of publication on climate change in six leading, generalist sociology journals across all available years through 2023. I also conduct what is, to my knowledge, the first review of climate change coverage in major sessions between 2002 and 2023 in American Sociological Association (ASA) Annual Meetings. Whereas journals and conference sessions provide broader measures of attention to climate change in sociology in the U.S., I also explore climate change research and teaching from a bottom-up perspective by examining faculty biographies and course offerings in the 2022 *U.S. News* 20 top-ranked U.S. sociology departments. Given the predominance of these departments in hiring and other outcomes (Burris, 2004; Wapman et al., 2022; also see Data and Method), the extent to which they attend to climate change in research and teaching may disproportionately influence coverage at national level.^4^

In this paper, I first summarize previous research on attention to climate change in research and teaching in sociology, as well as the absence of sociology and other social sciences in climate research and policymaking worldwide. I then describe my methods and results. I find notably low levels of climate change coverage across journal articles, conference sessions, faculty biographies, and course offerings, both in absolute terms and compared to other research topics. However, generalist sociology journals have published more articles on climate change in recent years. Finally, I discuss the implications of my findings, some possible explanations, and what sociologists, funders, journalists, and policymakers can do to increase socially informed understanding of and responses to climate change.

## Background

### Climate Change is Scarce in Sociology Courses and Journals

#### Climate Change in Sociology Courses

“Introduction to Sociology” textbooks and courses have reportedly dedicated little space to the environment and climate change. In 2000, Anthony Gidden’s *Sociology* devoted four out of 625 pages to climate change (Lever-Tracy, 2008).^5^ This is not an isolated example: an analysis of sociology textbooks published between 2000 and 2003 showed that on average they dedicated less than 3% of the text to environmental sociology, so presumably they discussed climate change even less, if at all (Lewis & Humphrey, 2005). A subsequent review suggested that not much had changed over a decade later (Liu & Szasz, 2019). Liu and Szasz (2019) found that the 11 best-selling sociology textbooks usually relegated environmental topics to the end, combined them in chapters with other topics (in which the environment always came last), and gave them only a few pages. Coverage dedicated to climate change was small, unsophisticated, and sometimes erroneous (by contrast, introductory economics textbooks dedicate more space to climate change; see Liu et al., 2019). Environmental topics are rare not only in sociology textbooks but also in syllabi for commonly offered introductory sociology courses (ASA, 2016).^6^ Lowney et al. (2017) found that environmental topics in general accounted for less than 1% of the “Introduction to Sociology” syllabi they collected from a range of universities, and appeared even more rarely, if at all, in “Social Problems” syllabi.

#### Climate Change in Sociology Journals

A similar pattern has emerged in coverage of climate change and the environment in generalist sociology journals, though here it appears to be increasing. When Lever-Tracy (2008) searched eight generalist sociology journals internationally for the terms “climate change,” “global warming,” or “greenhouse gas” between 2000 and 2005, there was not a single match in the titles or abstracts.^7^ A decade later, Koehrsen et al. (2020) conducted a similar search in internationally top-ranked sociology journals until 2018.^8^ These journals began to publish articles on climate change in 2008 but they remained a small percentage, representing less than 1% of these journals’ articles in 2017. By comparison, 13% that year concerned “social class” (Koehrsen et al., 2020).

In an analysis of articles in 30 top-ranked sociology journals from 1970 to 2006, Goodall (2008) found that 0.16% mentioned climate change or global warming in their titles, abstracts, or keywords. Goodall found similarly low rates in other social sciences (business and management, economics, and political science), and that the natural sciences overall had published more articles on climate change than these social sciences.

More recently, Scoville and McCumber (2023) observed that “climate change is among the most pressing problems of our time, yet it remains a marginal topic in sociology.” Looking at *American Sociological Review* (*ASR*) and *American Journal of Sociology* (*AJS*) articles between 2015 and 2020, they found that “only a single article…is centrally about climate change” (p. 9). They emphasized that sociological research on climate change *does* exist, but it is relegated to non-mainstream venues such as environmental sociology and science and technology studies journals. Their analysis of a broader set of sociology journals in 2020 using the Sociological Abstracts database similarly revealed that while a range of journals have published on climate change, they do so infrequently. As such, they “[uncovered] no unanalyzed hub for the sociology of climate change” (p. 22). Scoville and McCumber suggest that “climate silence” in mainstream sociology journals is a result not of disciplinary avoidance of “claims of nonhuman agency” and biophysical data, but of “existing research habits” (p. 20) (more on this in the Discussion).

### Sociology is Scarce in Climate Change Research and Assessments

Scholars have thus documented limited engagement with climate change within sociology. Furthermore, sociology *itself* is scarce in climate change research and major assessments. This may be unsurprising, as researchers have previously documented how the social sciences are underrepresented in general in climate change research (Goodall, 2008; Overland & Sovacool, 2020; Victor, 2015). In an analysis of research funding allocation in 37 countries, Overland and Sovacool (2020) found that hardly any social science research on climate change was conducted before 1990. Between 1990 and 2018, the natural sciences received 770% more research funding than the social sciences for climate-related research, and a mere 0.12% of all research funding went to the social science of climate mitigation (Overland & Sovacool, 2020).^9^

Sociology is particularly rare in national and global climate assessments such as the U.S. National Climate Assessment (NCA) and those of the IPCC. The scarcity of sociologist authors and cited sociological work contrasts especially with the dominance of economists in these assessments. For example, social scientists comprised 11% of authors of the 2018 NCA (Maxwell et al., 2022). Of these, 40% were economists and just 3% sociologists. Of the 14,000 journal references in the 2001 IPCC Third Assessment Report, 12% were from the social sciences, 39% of which were from economics. None came from sociology (Bjurström & Polk, 2011). The 468 coordinating, lead, and contributing authors of the 2014 IPCC AR5 Working Group III on climate mitigation pathways included 127 economists and one sociologist (Holm & Winiwarter, 2017). A separate analysis of the coordinating and lead authors in this group found that 28% were economists. The remaining social scientists represented 22% of authors and included no sociologists (Corbera et al., 2016). Victor (2015) reports that less than one third of the authors in Working Group II on Impacts, Adaptation, and Vulnerability that year were social scientists, and half of those were economists.

There is thus an observed lack of sociologists—and of the social sciences in general—in climate change research and assessments. Looking at their own discipline, sociologists have noted low engagement with climate change in sociology textbooks, introductory syllabi, and generalist journals. To my knowledge, reviews of climate change coverage in generalist journals have not yet examined years past 2020 or certain U.S. journals. Furthermore, such studies have not empirically examined coverage of climate change in conference proceedings or, at the departmental level, in faculty research foci and course offerings. These latter two measures are critical, as they provide insight into the training of undergraduate and doctoral sociology students—the sociologists of the future.

## Data and Method

I draw on four types of data in this study: research articles in leading generalist journals, ASA Annual Meeting major sessions, and faculty biographies and course offerings in the 20 top-ranked U.S. sociology departments. I take these forums to represent “mainstream” U.S. sociology or the “core” of the discipline (Perry 2023). Journal articles and ASA sessions can shed light on sociological discussion of climate change at the national level (and arguably international level, given the global prominence of U.S. journals; see Jacobs & Mizrachi, 2020). Previous research has examined inclusion in top sociology journals as a measure of a field’s centrality in sociology (e.g., Scott & Johnson, 2016). Faculty biographies and course offerings from top-ranked departments provide an on-the-ground sense of attention to climate change in departments that may wield disproportionate influence over the discipline nationally (Burris, 2004; Wapman et al., 2022; see Faculty Biographies and Course Offerings below).

In each dataset, I searched for climate change terms (climate, “global warming,” “greenhouse gas*,” fossil*, or carbon*) and, for comparison, a broader set of environment-related terms including climate change terms (“environment*,” “sustainabl*,” “pollut*,” “natur*,” green*, or “ecosystem*,” climate, “global warming,” “greenhouse gas*,” fossil*, or carbon*). Previous studies of sociological coverage of climate change have typically searched articles for the terms “climate change” and “global warming” (e.g., Scoville & McCumber, 2023). I added other climate-relevant search terms to be exhaustive; however, I obtained similar results when searching only for climate change and global warming. Though this article focuses on sociological engagement with climate change, I also conducted some exploratory analyses comparing coverage of climate change to that of other observed topics in sociology, such as race, health, and immigration. For select analyses, I cleaned the relevant data in R (e.g., lowercasing all letters, stemming terms, and removing common stopwords such as “and”) before conducting each search.

### Journal Articles

I used Web of Science and journal websites in December 2023 to search the journals *AJS*, *ASR*, *Annual Review of Sociology*, *Social Forces*, *Social Problems*, and *Socius*. Scott and Johnson (2017) identified *AJS*, *ASR*, *Social Forces*, and *Social Problems* as the “most prestigious mainstream journals” in sociology, according to Thomas Reuters 2014 Journal Citation Reports. All journals in my selection are U.S.-based, generalist journals that rank among the top 15 sociology journals by Journal Impact Factor (FHI) in 2023 (according to Journal Citation Reports by Clarivate).

I searched titles, abstracts, and keywords in each journal for climate terms for all years through December 2023. I then manually selected articles that pertained to climate change (e.g., ate”). I also calculated the percentages of all articles in these journals concerning climate change between 2006 and 2023. I examined “articles” as defined by Web of Science, to isolate research articles and exclude, for example, book reviews. Though climate change terms may be mentioned in the body of an article, I assume that mentioning them in the title, abstract, or keywords indicates that the article is substantially about climate change. For example, Scoville and McCumber (2023) searched all articles in *ASR* and *AJS* between 2015 and 2020 containing “climate change” or “global warming” anywhere in the text. After qualitatively coding each article, they found that only one out of 21 was centrally about climate change (Entwisle et al., 2020), which matches my results for that time period for *AJS* and *ASR* from searching titles, abstracts, and keywords.

### ASA Annual Meeting Major Sessions

ASA, the U.S. national professional membership association for sociologists, hosts an Annual Meeting that draws thousands of attendees from a range of subfields. This central gathering thus reflects mainstream sociology. Though these conferences consist of hundreds of presentations and poster sessions, I limited my search for climate-related content in the titles and descriptions of major sessions, including presidential addresses, presidential panels, and plenary sessions, because these presumably draw larger, broader audiences. Presidential and plenary sessions are “chosen by the ASA president” as “of interest to a large population of the membership,” and sometimes no other programming is scheduled during plenary sessions (e.g., ASA, 2017).

### Faculty Biographies and Course Offerings

I examined faculty and course offerings in the *U.S. News* top-20 departments (as of December 2022) because of the disproportionate influence that these departments may have on sociological research and education across the country. Indeed, sociologist Burris (2004) highlighted that the “prestige of the department in which an academic received a PhD consistently ranks as the most important factor in determining the employment opportunities” of doctoral students across disciplines. He found that graduates from the top 20 sociology departments accounted for 70% of faculty hired in 94 PhD-granting departments. If this trend has continued, graduate students trained in the 20 top-ranked sociology departments today may disproportionately represent future sociology faculty across the U.S. The lack of climate change coverage in top-ranked departments may thus have implications for trends in research and teaching in sociology departments across the country.

Burris’s observations are also important because of the impacts of university and department prestige beyond the academy. For example, prestigious academic affiliations improve paper acceptance rates, publication rates, access to resources, citations and attentions, and the likelihood of receiving awards (Wapman et al., 2022). More prestigious departments may thus disproportionately shape the spread of ideas both in and beyond academia.

#### Faculty Biographies

With a research assistant in January 2023, I compiled the keywords and biographies from assistant, associate, and full faculty university webpages in the top-20 departments. This yielded a sample of 562 professors (107 faculty did not include keywords in their webpage). Faculty biographies typically consisted of a 1–2-paragraph description. When this was not available, I took a description from another university webpage (e.g., a research center at the same university, or from a previous university appointment) or a personal website. I was not able to find online biographies for 13 faculty. In these cases, I examined their CVs for mentions of climate keywords. Of faculty with online biographies available, 102 mentioned climate and environment-related terms. I manually reviewed these faculty and excluded irrelevant cases (e.g., using terms such as “social environment” or “natural language processing”).

A limitation of this method is that faculty webpages, personal websites, and CVs may not be up to date, concealing more recent climate change research and underestimating attention to climate change in these departments. However, if much of sociological climate change research is unpublished, this would suggest that this research is only recently emerging in these departments. Furthermore, these sources are important because they reflect publicly available information about these faculty that audiences such as prospective graduate students might see. Students interested in researching climate change or other environmental topics may not pursue programs lacking faculty with these interests stated in their online profiles. On the other hand, these estimates may overestimate the extent to which faculty research climate change, since I include faculty whose profiles only mention these keywords peripherally but whose research may not focus substantially on climate change.

#### Course Offerings

I analyzed courses offered in 18 of the top-20 departments over four years, from Fall 2019 to Spring 2023 (approximately eight semesters for each department, depending on university calendars). I examined a subset of courses from this period for the remaining two departments for which I was not able to obtain the full sample. Examining a four-year period helps account for courses that are not offered frequently. With a research assistant, I compiled course listings in multiple ways: publicly available online; provided by department staff; and one public records request. I rely on course titles in this analysis because some departments were not able to include course descriptions in the data they provided, and sometimes course descriptions were not included in public listings (see Supplementary Information for more detail). A limitation of examining course titles alone is that this may not account for instances where climate change is not mentioned in a title but is included in the syllabus, for example in an Introduction to Sociology course. I thus supplemented this analysis by searching for climate keywords in *Open Syllabus Analytics*, which maintains a database of 124,375 sociology syllabi in the U.S.

## Results

### Journal Articles

**Fig. 1:**
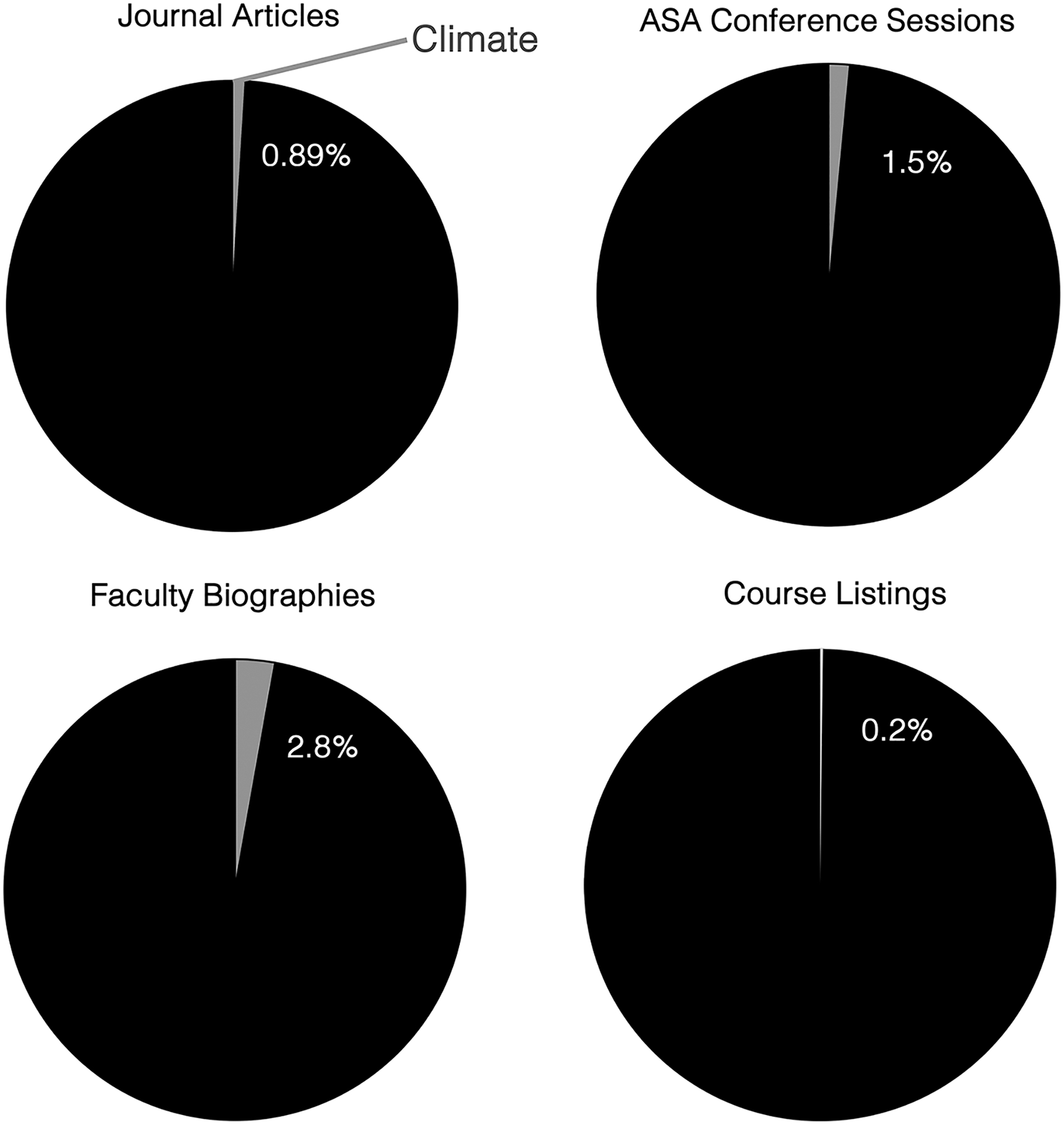
Climate change coverage in journal articles, conference sessions, faculty biographies, and course listings. Note. ASA conference sessions include thematic sessions (*N* **=** 599)

Overall, coverage of climate change is relatively limited in my selection of leading, generalist sociologist journals (Table 1). I narrowed my analysis to 2006–2023 because only one article centrally regarded climate change before this point. Between 2006 and 2023, 0.35%, 0.14%, 0.29%, and 0.99% of all articles in *AJS*, *ASR*, *Social Problems*, and *Social Forces*, respectively, focused on climate change. The *Annual Review of Sociology* (2.31%) and *Socius* (1.69%), a newer journal launched in 2016, published higher percentages. Overall, 0.89% of all articles published between 2006 and 2023 in these six generalist journals centrally concerned climate change (Fig. 1).^10^

Since its establishment in 1895, *AJS* published two research articles on climate change, in 2020 and 2023 (Entwisle et al., 2020; Wetts, 2023). *ASR* published its first climate-central article in 2023 (Jorgenson et al., 2023). This finding aligns with Scoville and McCumber’s (2023) search of 2015–2020 articles in these journals.

*Annual Review of Sociology* has published nine articles on climate change since 2011 (Arcaya et al., 2020; Beckfield & Evrard, 2023; Centeno et al., 2015; Dietz et al., 2020; Gauchat, 2023; Hunter et al., 2015; Klinenberg et al., 2020; Kraly et al., 2023; Rudel et al., 2011). Some of these focus on climate change; others primarily refer to it as an example of a broader phenomenon. This higher number of articles is perhaps unsurprising, as a key role of this journal is to identify new areas for research. *Social Forces* and *Socius* have published the greatest number of climate-related articles since 2006. Over 80% of these were published after 2018.

I searched only for research articles, but *AJS* and *Social Forces* also publish book reviews. Though these are not accorded the same prestige as research articles, they provide a sense of sociological books being released about climate change and interest in reviewing them. Since their founding, *AJS* and *Social Forces* have respectively published ten and four reviews of books related to climate change. Interestingly, the earliest reviews, from the 1920s and 1940s, discussed the social relevance of historical climatic changes, but did not necessarily refer to the current anthropogenic climate crisis. Most reviews were published since 2016.

Searches for other topics such as health, crime, immigration, and education yielded more hits in each journal compared to climate keywords. For example, *ASR* has 130 articles with “education” in the abstract compared to six with any climate keywords. This comparison does not account for accuracy: only one of those six was actually about climate change, so perhaps not all of the articles with “education” in the abstract are primarily about education. But it gives an order-of-magnitude sense of the relative scales at which different social topics are covered.

**Fig. 2:**
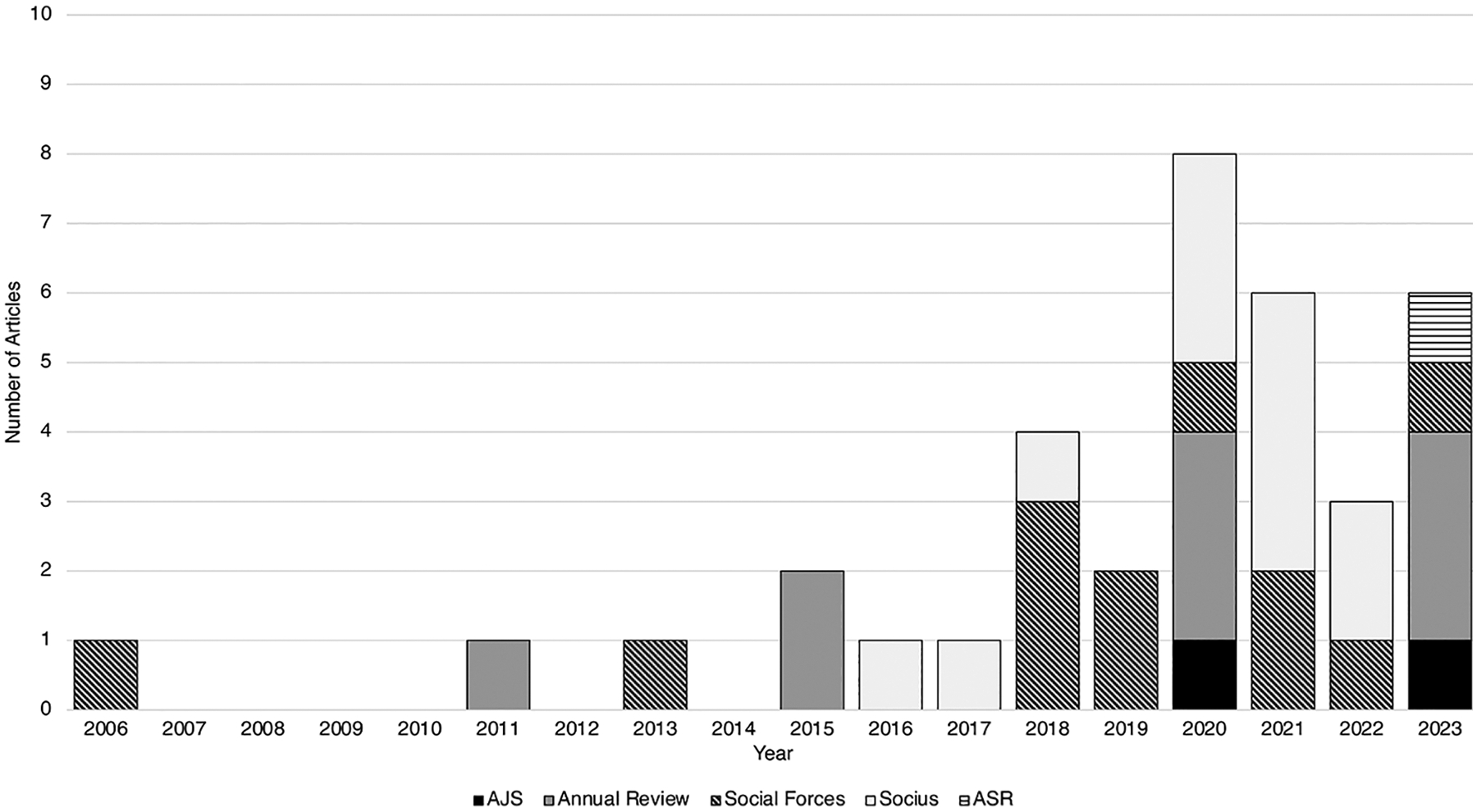
Climate change research articles in U.S. sociology journals 2006–2023

Though these results point overall to relatively limited discussion of climate change, these journals began to publish more articles on the topic in recent years, as illustrated in Fig. 2. From 2006 to 2014, no more than one journal in this selection published an article on climate change in a given year. From 2020 onward, multiple journals published on this topic each year, with all journals except *Socius* doing so in 2023.

### ASA Annual Meeting Major Sessions

Table 2 shows the number of ASA Annual Meeting major sessions that mentioned climate change in 2002–2023. Two out of 171 major sessions, or 1.17%, mentioned climate change, including presidential addresses, presidential panels, and plenary sessions. The 2016 Annual Meeting hosted a presidential panel on “Climate Change and Social Movements,” and the 2022 Annual Meeting hosted a presidential panel on “Climate Change, Disaster, and Displacement.”^11^ When thematic sessions (and the Ford Panels in 2004) are included in the sample, 1.5% or nine out of 599 sessions mentioned climate change (Fig. 1). Climate change or other environmental topics were not the theme of any conference.

### Faculty Biographies

Of 562 faculty in 20 departments, 2.8% (16) mentioned climate change terms in their online biography or CV as of January 2023 (Table 3; Fig. 1). Nine departments had no faculty members who did so. When I broadened my search to include all environmental terms (which include climate change keywords), a higher 5.5% (31 of 562) of faculty met the criteria.

### Course Offerings

I searched mentions of climate change terms in 8,243 undergraduate and graduate course titles over eight semesters across four years, from Fall 2019 to Spring 2023, in the 20 top-ranked departments (with partial course offerings for two departments; see Supplementary Information). Table 4 summarizes how many relevant courses were offered by each department.

0.2% of all courses (21 out of 8,243) focused on climate change (Table 4; Fig. 1), while 1.4% concerned environmental topics in general. An exploratory analysis in Table 5 suggests that courses on environmental topics—which included climate change—were offered less frequently than those on other topics such as race, inequality, and health. In a review of the top 100 terms in course titles (after removing common stopwords such as “and,” as well as non-substantive terms such as “seminar” or “sociology”), I find that race (403 mentions), inequality (316), gender (262), politics (249), culture (242), and health (240) are within the top 10. “Environment” places 67th, and there are no climate-change-related terms in the top 100.

I supplemented my analysis of course titles by searching climate change keywords in *Open Syllabus Analytics*, which maintains a database of 124,375 sociology syllabi in the U.S.^12^ In 2022, climate change and global warming were mentioned in 1.5% and 0.2% of syllabi, respectively, far less often than topics such as education (22.7%), family (26.9%), health (11.7%), crime (11.7%), and immigration (8.9%).

## Discussion

Sociologists have repeatedly called for further sociological research and teaching on climate change over at least the past 15 years (Lever-Tracy, 2008). Some have documented relatively low rates even when compared to other important social problems (Koehrsen et al., 2020). In this paper, I addressed the question: What is the status of engagement with climate change in U.S. sociology today? To do so, I updated and expanded analyses of leading generalist journals to 2023, and supplemented them with analyses of ASA major sessions and faculty biographies and course offerings in 20 top-ranked sociology departments.

Overall, I find limited attention to climate change across these forums. It is mentioned in just 0.89% of journal artices, with almost nonexistent coverage before 2006. However, generalist sociology journals have begun to publish more articles on climate change in recent years, particularly since 2018. *AJS* and *ASR* have collectively published three articles on climate change since their founding, two of them in 2023. Though these numbers are low, they suggest a possible trend toward increased coverage of climate change in these journals. Meanwhile, at ASA Annual Meetings, 1.17% of major sessions substantially discussed climate change between 2002 and 2023. This percentage only increases to 1.5% when hundreds of thematic sessions are included. Two conferences mentioned climate change in their major sessions, but it was not the theme of any conference. These findings align with recent studies of sociological publications on climate change, such as Scoville and McCumber (2023), and anecdotal discussion of ASA Annual Meetings by Norgaard (2018).

I also extend previous analyses by examining attention to climate change at the ground level in 20 top-ranked sociology departments in the U.S. Of 562 faculty, 2.8% mentioned climate change terms in their webpage or CV. Nine departments had no faculty members who mentioned climate change. 5.5% of faculty mentioned environmental terms (including climate terms) more generally. Of the 8,243 courses offered in these departments from Fall 2019 to Spring 2023, 0.2% concerned climate change, and 1.4% mentioned environmental topics more generally.

These findings are concerning, especially given the disproportionate influence these journals and departments may have on the discipline at large (Burris, 2004; Jacobs & Mizrachi, 2020). Sociologist Rebecca Elliott (2018) writes that “climate change is present for sociology; to ignore it is to ignore the world we currently inhabit” (p. 304). Not only is climate change clearly an appropriate topic for sociological study, but it is worsening; it poses unique existential issues for humanity; and it has been documented by climate scientists, lawmakers, journalists, and activists for over half a century. As activist Bill McKibben has written, climate change differs from other social problems because “it won’t stand still” and can have irreversible effects (McKibben, 2017).

Referring to higher education more broadly, Thierry et al. (2023) warn that despite the mounting impacts of climate change, “most academics continue to operate according to ‘business-as-usual’” and offer limited teaching and research on climate change. “Such passivity increases the risk of climate impacts so severe as to threaten the persistence of organized society, and thus [higher education institutions] themselves,” they write. Universities sometimes may even undermine climate action, for example by investing endowments in fossil fuels, or partnering with fossil-fuel companies to conduct research supporting fossil-fuel production or technologies promoted by that industry such as carbon capture and storage (Almond et al., 2022; Barron et al., 2023; Hiltner et al. 2024; Supran & Franta, 2017).

What are the implications of limited research and teaching on climate change in sociology? A lack of courses about climate change fails to establish it as a topic relevant to sociological inquiry, may limit students’ knowledge about a current, worsening, and existential phenomenon, and, in the case of graduate students, may fail to inspire related research or prepare future faculty with expertise on climate change. Furthermore, sociologists, and social scientists in general, may be ignoring a major channel of potential research funding. Scholars also worry that a lack of sociological inquiry (and of social science in general) may risk marginalizing sociology in the wider world of climate research (Overland & Sovacool, 2020), international assessments such as those by the IPCC, and policymaking, with implications for how climate change is framed and responded to as a social problem.

Indeed, social scientists have long lamented the dominance of the natural sciences in climate change research (Castree et al., 2014; Overland & Sovacool, 2020). Though the natural sciences have thoroughly documented the changing climate and its implications for the biophysical world, and have modeled its future effects, they cannot fully explore questions such as how humans contribute to, are impacted by, and can respond to these changes. Furthermore, the apparent dominance of economists in the small share of social science research on climate change and authorship in climate assessments (e.g., Holm & Winiwarter, 2017) may promote a limited social scientific perspective on climate change (Garth & Roberts, 2022; Oreskes & Conway, 2023; Popp Berman, 2022).

### Is the Tide Turning?

Some scholars have suggested that sociology has increasingly turned its attention to the climate crisis, often noting that the ASA Council convened the *ASA Task Force on Sociology and Climate Change* in 2010 to synthesize sociology’s contributions to climate change research, culminating in the report *Climate Change and Society: Sociological Perspectives* (Dunlap & Brulle, 2015). The National Science Foundation also funded a workshop on “Sociological Perspectives on Global Climate Change” in 2008, and ASA helped disseminate the final report (Nagel et al., 2010). Did these gatherings spark broader sociological interest in climate change? We can only speculate, but this study suggests limited attention *overall* to climate change across generalist journals, conference sessions, and faculty research and course offerings in top-ranked departments.

However, I also observed an increase in the absolute numbers of journal articles and conference proceedings on climate change in the 2010s. The Climate Social Science Network was launched in 2020 and includes sociologists. An ASA report on faculty job advertisements observes that the “top areas desired by employers seem to reflect current issues, including those related to race relations, criminal justice, and *climate change*” (2023, p. 5, my emphasis). However, it notes that “the areas of specialization of interest to employers do not fully align with the research interests specified by ASA student members” (p. 5). Whereas 8.3% of advertised positions called for a specialization in environmental sociology—a percentage that has increased since 2019—only 5.3% of ASA student members identified it as an interest area (ASA, 2023). This finding points to the importance of understanding bottom-up determinants of sociological interest, such as at the departmental level. I also found that flagship journals *AJS* and *ASR* published articles mentioning climate change in 2023, and other generalist journals have published an increasing number of articles, in particular since 2018. Furthermore, in 2020 the *Annual Review of Sociology* published articles outlining directions for future research on climate change (Dietz et al., 2020; Klinenberg et al., 2020).

### Why is Attention to Climate Change Limited in Sociology?

Why have the U.S. sociological forums in this study neglected to substantially attend to climate change? This pattern is puzzling given sociology’s attention to important social problems. Answers are necessarily speculative given the absence of empirical research into this question (see What Is to Be Done? below for suggested future research). Climate change is a unique case because scant attention does not appear to reflect *epistemic exclusion*, as with feminist sociology in the mid-20th century (Mezey, 2020).^13^ Indeed, sociologists as a group are likely disproportionately liberal and concerned about climate change (e.g., see Duarte et al., 2015, on the liberal leanings of psychologists).

Scholars have posed a range of possible explanations. Some turn to the foundations of sociology during the Industrial Revolution and accompanying major shifts in social structure and cultural values (Grundmann & Stehr, 2010). Some argue that early sociologists such as Émile Durkheim assumed a strict divide between society and “nature” (Catton & Dunlap, 1980; Norgaard, 2018; Scoville & McCumber, 2023), though others note that early theorists such as Karl Polanyi and Karl Marx exhibited environmental concern (Brechin & Fenner, 2017; Foster, 1999; Klinenberg et al., 2020).^14^ Grundmann and Stehr (2010) suggest that the political nature of climate change and methodological differences with the natural sciences may also play a role.

In their article on “climate silence” in sociology, Scoville and McCumber (2023) suggest that “the largest barriers to bringing climate change to the center of the broader discipline may simply be *existing research habits*” (p. 20, my emphasis). In other words, sociologists perhaps just aren’t used to climate change as a research topic yet. Available career paths may also shape the research foci of graduate students and junior faculty. Universities often require that new faculty publish frequently in the area of specialization specified in a job advertisement. If environmental sociology or climate change are not mentioned in job listings, graduate students may be less motivated to pursue these topics, and junior faculty will find it difficult to devote energy to these areas while trying to earn tenure. But, as I note above, it appears that employers are increasingly calling for applicants focused on climate change (ASA, 2023).

In a similar vein, perhaps sociology has not had as much time to define anthropogenic climate change as a coherent field, like race or gender. Dunlap and Brulle (2015) suggest that sociological input on climate research and policymaking—in IPCC assessments for example—may be limited because “sociological research on climate change emerged only recently (beginning in the 1990s and increasing rapidly in the past decade), is spread across a wide variety of academic journals and books, and is neither easily identifiable by nor accessible to the wider intellectual community” (p. 2). For example, *ASR* has published some articles on fracking—a controversial method of extracting natural gas and a contributor to climate change—but they did not appear in my search results, because they did not include mentions of climate change in the title, abstract, or keywords.^15^ On the other hand, the “not enough time” explanation on its own is insufficient because, again, climate scientists, journalists, activists, and lawmakers have raised the alarm about climate change for decades (Bell, 2021; Oreskes & Conway, 2011).

### What is to Be Done?

#### Recommendations for Practice

Where do sociologists go from here? In this paper, my intention is not to dissect the research of individual scholars and cast blame (e.g., in my analysis of 20 departments) but rather to examine sociology in the U.S. at large and to stimulate a conversation about the reasons for and ramifications of sociology’s relative silence on climate change.

That said, going forward, sociologists can consider how climate change relates to their particular research lens—and, more importantly, promote attention to climate change in the education of students and future sociologists, including climate change in undergraduate and graduate courses, and encouraging relevant research by graduate students. A proposal for future research directions in sociology regarding climate change is beyond the scope of this article but is thoroughly discussed elsewhere for interested readers (e.g., Bhatasara, 2015; Davidson, 2022; Dietz et al., 2020; Elliott, 2018; Grundmann & Stehr, 2010; Klinenberg et al., 2020; Mezey, 2020; Zehr, 2015). Generalist sociology journals’ editorial staff and conferences may prioritize coverage of climate change; ASA, for example, can dedicate an Annual Meeting to the environment and climate change.

As for teaching, Mezey (2020), former president of the Society for the Study of Social Problems, recommends either offering a full course on climate change or bringing it into existing courses such as Introduction to Sociology. Pelton (2022) thoroughly discusses teaching about the environment and climate change, recommending McNall and Szasz’s 2014 *TRAILS* resource, “Teaching the Sociology of Climate Change,” which suggests student learning outcomes, lesson plans, and assessments. The ASA Teaching and Learning in Sociology section can provide additional guidance on climate change education. Teaching is especially important for laying the groundwork to educate younger generations and future sociologists on this topic. Sociology departments may also consider collaborating with environmental studies and climate change centers at their universities, for example through coordinating workshops, joint course offerings, and cross-departmental appointments.

While sociologists may do more to research and teach about climate change, funders, journalists, and policymakers may do more to foster and integrate social science perspectives. Research funders may allocate more to social-science climate research. As noted above, Overland and Sovacool (2020) found that a mere 0.12% of all climate research funding went to social science mitigation research.

Journalists and media groups may dedicate more space to the social dimensions of climate change. A report by Carbon Brief shows that of the top 10 climate research papers featured in the news and social media in 2023 (McSweeny and Tandon, 2024), only two concerned the human dimensions of climate change (with similar results in 2022 and 2020) (McSweeny, 2021; McSweeny and Tandon, 2023). This suggests that natural-science perspectives are predominantly featured in mainstream media coverage of climate change. Finally, international assessments such as those by the IPCC and policymakers may make a more concerted effort to incorporate the social sciences in climate research and policymaking.

#### Recommendations for Future Research

Sociologists may further the present study by empirically examining *why* attention to climate change is limited in sociology, and related questions such as how the study of climate change and other environmental topics is perceived within the broader discipline. For example, Perry (2023), examining the marginalization of the sociology of religion, surveyed sociology faculty and trainees about their evaluations of religion and other subfields. Researchers interested in investigating why climate change research is relatively scant in sociology may conduct interviews with departmental chairs, journal editors, conference organizers, and other potentially influential figures in the discipline, as well as examine trends in sociology journals, job listings, dissertation topics, or faculty biographies and course listings from a broader sample of universities and time period. Such research may shed light on the mechanisms by which topics become central objects of study in sociology, and, in the case of climate change, sociology’s approach to examining the relationship between nature and society.

Scholars may also compare the degree and nature of attention to climate change in sociology to those in other social science disciplines, such as economics and psychology. They may examine the relative presence of various social sciences in climate policymaking circles, advocacy organizations, and assessments such as those of the IPCC, and how this shapes the production of climate knowledge and construction of climate change as a social problem. Preliminary findings suggest that economists are more represented than other social sciences in climate policymaking—what are the implications of this? (Victor, 2015).

The dynamics between research and policymaking are not necessarily linear or unidirectional, however. For example, more research on climate change in a discipline does not necessarily mean it will be more represented in policymaking or the IPCC, and more representation of a discipline in an organization does not guarantee that the discipline’s perspective will substantially shape the final product. Scholars may further investigate these dynamics to understand what factors shape the representation of sociology and other social sciences in climate policymaking, and their consequences.

## Conclusion

The underrepresentation of the social sciences, particularly sociology, in climate research and policymaking raises the question of the extent to which sociology itself attends to climate change. In this work I examined attention to climate change in sociology journals, conference sessions, faculty biographies, and course offerings in the U.S. I found notably limited engagement with climate change across these data sources, even when compared to attention given to other important social topics. These deficits are particularly concerning for their implications for the training of future generations of sociologists coming of age in a changing climate and the availability of frames to understand and respond to climate change as a social problem.

Sociologists may integrate climate change into existing or new courses and foster climate change research among students and faculty; funders and philanthropists may allocate more climate research funding to the social sciences; journalists may highlight social perspectives on climate change; and policymaking circles such as the IPCC may include more social scientists in their ranks. Future research may further examine the reasons for sociology’s limited attention to climate change, examine trends in climate change research and education in other social science disciplines, and investigate the dynamics between climate research and policymaking.

## Supplementary Material

Supplementary Material 1

## Figures and Tables

**Table 1:** Climate change research articles published in U.S. sociology journals 2006–2023

Journal	Total Articles	Mention Climate Change	
		#	%
*American Journal of Sociology*	572	2	0.35%
*American Sociological Review*	708	1	0.14%
*Annual Review of Sociology*	389	9	2.31%
*Social Problems*	699	2	0.29%
*Social Forces*	1,212	12	0.99%
*Socius* ^a^	708	12	1.69%
**Total**	**4,288**	**38**	**0.89%**

Note. Though the total articles obtained from Web of Science for each journal may not be fully accurate, I do not expect possible inaccuracies to substantially affect these results

a I calculated the total *Socius* articles using Web of Science for 2020–2023 and the *Socius* website for 2016–2019, as *Socius* was launched in 2016 but Web of Science did not include all articles before 2020. A search of climate terms for all articles published in 2016–2023 using the *Socius* website revealed no additional articles related to climate change

**Table 2:** Climate change in the ASA Annual Meeting major sessions 2002–2023

Year	Theme	Mention Climate Change
2002	The Question of Culture	0
2003	Public Sociologies	0
2004	Comparative Perspectives, Competing Explanations Accounting for the Rising and Declining Significance of Sociology	0
2005	Great Divides: Transgressing Boundaries	0
2006	Is Another World Possible? Sociological Perspectives on Contemporary Politics	0
2007	Worlds of Work	0
2008	The New Politics of Community	0
2009	Toward a Sociology of Citizenship	0
2010	Social Convict: Multiple Dimensions and Arenas	0
2011	Allocation Processes and Ascription	0
2012	Real Utopias: Emancipatory Projects, Institutional Designs, Pos-sible Futures	0
2013	Interrogating Inequality: Linking Micro and Macro	0
2014	Hard Times: The Impact of Economic Inequality on Families and Individuals	0
2015	Sexualities in the Social World	0
2016	Rethinking Social Movements: Can Changing the Conversation Change the World?	1
2017	Culture, Inequalities, and Social Inclusion across the Globe	0
2018	Feeling Race: An Invitation to Explore Racialized Emotions	0
2019	Engaging Social Justice for a Better World	0
2020	Power, Inequality, and Resistance at Work	0
2021	Emancipatory Sociology: Rising to the Du Boisian Challenge	0
2022	Bureaucracies of Displacement	1
2023	The Educative Power of Sociology	0

Note. Number of plenary sessions and presidential addresses and panels that mention climate changerelated terms. This table does not include thematic sessions

**Table 3:** Faculty biographies mentioning climate change in sociology departments

University	Total Faculty	Mention Climate Change	
		#	%
Berkeley	30	3	10%
NYU	32	3	9.4%
Brown	26	2	7.7%
Yale	17	1	5.9%
Cornell	21	1	4.8%
Chicago	24	1	4.2%
Northwestern	25	1	4%
Harvard	27	1	3.7%
UW-Madison	28	1	3.6%
Princeton	29	1	3.4%
Columbia	29	1	3.4%
Michigan	39	0	0%
Stanford	22	0	0%
UCLA	38	0	0%
UNC-Chapel Hill	25	0	0%
UT-Austin	42	0	0%
Duke	21	0	0%
University of Pennsylvania	25	0	0%
Indiana University Bloomington	27	0	0%
OSU	35	0	0%
**Total**	**562**	**16**	**2.8%**

Note. Number and percentage of faculty online biographies or CVs that mention climate change. Online biographies were accessed in January 2023

**Table 4:** Courses on climate change in the sociology departments

University	Total Courses	Mention Climate Change	
		#	%
Berkeley	427	4	0.9%
NYU^b^	269	3	1.1%
Harvard	340	2	0.6%
Northwestern	330	2	0.6%
University of Pennylvania	332	2	0.6%
Indiana University Bloomington	435	2	0.5%
Princeton	211	1	0.5%
Michigan	464	1	0.2%
Stanford	744	1	0.1%
Chicago	432	1	0.2%
Yale	302	1	0.3%
Brown	284	1	0.4%
UCLA	754	0	0.0%
UNC-Chapel Hill	274	0	0.0%
UW-Madison	435	0	0.0%
Columbia^b^	235	0	0.0%
UT-Austin	630	0	0.0%
Duke	525	0	0.0%
Cornell	238	0	0.0%
OSU	580	0	0.0%
**Total**	**8243**	**21**	**0.2%**

Note. Total and percentage of undergraduate and graduate course titles offered in Fall 2019–Spring 2023 by each department mentioning climate change.

b I examine partial course offerings for this time period for NYU and Columbia (see Supplementary Information)

**Table 5:** Course topic frequency

Topic	Count	%
Race and Ethnicity	687	8.3%
Inequality	586	7.2%
Health and Medicine	366	4.4%
Gender	338	4.1%
Crime	261	3.2%
Immigration	171	2.1%
Environment	119	1.4%

Note. Keywords for non-environmental topics included: race and ethnicity (race*, racism, ethnic*), inequality (stratif*, ineq*, poverty), health and medicine (health, medic*), gender (gender, sex*), crime (crim*), immigration (migra*, immigra*). Environmental topics include the same keywords described under Data and Method

## Data Availability

After acceptance for publication, the data underlying this article can be made publicly and freely available in the ICPSR Data Archive at the University of Michigan.

## Footnotes

1 Sociologists have similarly expressed concern about sociology’s lack of engagement with environmental topics in general. Catton and Dunlap (1980) attributed this phenomenon to the “Human Exemptionalist Paradigm” (HEP), the Western worldview that humans are “fundamentally different from all other creatures, masters of their own destiny in a vast world with (seemingly) unlimited resources that provide the basis for unlimited technical progress” (Scoville & McCumber, 2023, p. 3). A lack of research on climate change may be one consequence of this paradigm. At the same time, Scott and Johnson (2017) found that environmental sociology has had a growing presence in top U.S. sociology journals between 1970 and 2014, in particular after 1990. They suggest that its growing acceptance by mainstream sociology was “critically facilitated by [environmental sociology’s] increased attention to core sociological concerns of stratification and inequality” (p. 1).

2 Of course, sociologists have examined climate change in the subfields of environmental sociology and science and technology studies (Scoville & McCumber, 2023), but climate change appears peripheral to the “core of the discipline” (Perry, 2023), which I measure in this study with leading generalist journals, ASA Annual Meeting major sessions, and faculty research and teaching in the 2022 U.S. News 20 top-ranked departments.

3 This is not the first time that sociologists have challenged the state of the field. Jacobs and Mizrachi (2020) detail how scholars have historically critiqued the “dominance of quantitative over qualitative research” and “under-representation of gender and race as topic, and women and minorities as authors” in leading sociology journals (Brown & Gilmartin, 1969; Coser, 1975; Ferree et al., 2007; Jacobs, 2007; McNamee et al., 1990; Ward & Grant, 1985; Wiley & Zelditch, 1979). Perry (2023) examines the marginalization of the sociology of religion. Scholars have also worked to counter such trends—for example, Mezey (2020) describes how “feminist scholars took important career risks by advocating for a feminist lens to shift the theoretical and empirical center of sociology” (Stacey & Thorne, 1985).

4 U.S.-based journals and the ASA Annual Meetings of course feature research by scholars around the world (e.g., Jacobs & Mizrachi, 2020). For simplicity, and since they are based in the U.S., I refer to “U.S. sociology” or “national-level” coverage of climate change in sociology.

5 Giddens later published *The Politics of Climate Change* in 2009. Interestingly, according to Grundmann and Stehr (2010), in this book Giddens “makes very little use of sociological frameworks.…Instead, he applies tools from political science…and public policy” (p. 906).

6 A 2016 survey by the American Sociological Association (ASA) found that nearly 100% of departments offered an “Introduction to Sociology” course “generally every term,” and a “Social Problems” course either every term or at least once an academic year.

7 Lever-Tracy (2008) searched titles and abstracts in *Acta Sociologica*, *American Journal of Sociology*, *American Sociological Review*, *British Journal of Sociology*, *Current Sociology*, *International Sociology*, *Journal of Sociology*, and *Sociology*, as well as the influential Marxist journal *New Left Review*.

8 Koehrsen et al. (2020) searched keywords, titles, and abstracts in a different set of journals than Lever-Tracy (2008): *American Journal of Sociology*, *American Sociological Review*, *Annual Review of Sociology*, *British Journal of Sociology*, *Current Sociology*, *European Sociological Review*, *Sociology*, and *The Sociological Review* (see Method on page 743).

9 In addition, according to Norgaard (2018), the International Social Science Council reported that 3% of publications on climate change are by sociologists (ISSC & UNESCO, 2013).

10 Since these journals largely did not start publishing articles about climate change until the late 2010s, there is not enough data to infer trends in the percentage of climate articles in these journals over time.

11 Norgaard (2018) notes, “as of this writing in 2017, climate change has only once been the subject of a plenary session at the American Sociological Association meetings (in 2014), was not mentioned in a Presidential address until 2016,” but I do not see any mention of climate change in major sessions in 2014.

12 Open Syllabus is a non-profit research organization that collects and analyzes millions of syllabi. It primarily obtains syllabi from publicly accessible university websites and receives a small portion from faculty submissions. It updates its dataset twice per year. More information about its method and data can be found on its website: https://blog.opensyllabus.org/about-os.

13 Thank you to Dr. Erin Cech for this observation.

14 Scoville and McCumber (2023) describe how Durkheim distinguished “social facts” from biological and geographical factors. However, Elliott (2018) argues that “any nature–social divide in social theory has been unproductive as best, delusional at worst, belied dramatically by the very notion of an Anthropocene.”

15 Thank you to Chalem Bolton and Erin Ice for this observation.
