# Supplementary material for: Limited Attention to Climate Change in U.S. Sociology
Source: Am Sociol. Author manuscript; Available in PMC 2025 Aug 29. (PMC12393807; doi:10.1007/s12108-024-09624-4)
Supplement: Supplementary Material 1 [file NIHMS2076286-supplement-Supplementary_Material_1.docx]

**Supplementary Information**

**Additional Information on Compiling Course Listings**

To compile course listings, I reached out to all 20 sociology departments in my sample to inquire whether they could share listings with me for Fall 2019 to Spring 2023. Staff members at eight departments generously emailed me their course offerings for this period. UCLA Information Practices provided me with listings in response to my request under the California Public Records Act at UCLA.

For the remaining 11 departments, I reached out to each university’s registrar. Some offered helpful advice on how to track down course listings, but none provided the requested data. I worked with a research assistant to compile these listings. Gathering them proved arduous, as universities varied in (1) whether they shared historical course listings publicly and (2) what format they shared them in (e.g., some only included course titles, without descriptions). Despite efforts to circumnavigate these barriers (e.g., searching departmental webpages using Wayback Machine), we were not able to compile all course listings for the full time period for NYU and Columbia. We specifically were unable to track down graduate sociology courses at Columbia for Spring and Summer 2020, and Spring, Summer, and Fall 2022, and undergraduate and graduate sociology courses at NYU for Fall 2019 to Spring 2021. The courses we were able to compile from Columbia and NYU are included in the full sample for analysis.
